# An Atypical Mitochondrial Carrier That Mediates Drug Action in *Trypanosoma brucei*

**DOI:** 10.1371/journal.ppat.1004875

**Published:** 2015-05-06

**Authors:** Juan P. de Macêdo, Gabriela Schumann Burkard, Moritz Niemann, Michael P. Barrett, Henri Vial, Pascal Mäser, Isabel Roditi, André Schneider, Peter Bütikofer

**Affiliations:** 1 Institute of Biochemistry and Molecular Medicine, University of Bern, Bern, Switzerland; 2 Graduate School for Cellular and Biomedical Sciences, University of Bern, Bern, Switzerland; 3 Institute of Cell Biology, University of Bern, Bern, Switzerland; 4 Department of Chemistry and Biochemistry, University of Bern, Bern, Switzerland; 5 Wellcome Trust Centre for Molecular Parasitology, Institute of Infection, Immunity and Inflammation, and Glasgow Polyomics, College of Medical, Veterinary and Life Sciences, University of Glasgow, Glasgow, United Kingdom; 6 Dynamique Moléculaire des Interactions Membranaires, CNRS UMR 5235, Université Montpellier II, Montpellier, France; 7 Swiss Tropical and Public Health Institute, Basel, Switzerland; 8 University of Basel, Basel, Switzerland; University of Dundee, UNITED KINGDOM

## Abstract

Elucidating the mechanism of action of trypanocidal compounds is an important step in the development of more efficient drugs against *Trypanosoma brucei*. In a screening approach using an RNAi library in *T*. *brucei* bloodstream forms, we identified a member of the mitochondrial carrier family, TbMCP14, as a prime candidate mediating the action of a group of anti-parasitic choline analogs. Depletion of TbMCP14 by inducible RNAi in both bloodstream and procyclic forms increased resistance of parasites towards the compounds by 7-fold and 3-fold, respectively, compared to uninduced cells. In addition, down-regulation of TbMCP14 protected bloodstream form mitochondria from a drug-induced decrease in mitochondrial membrane potential. Conversely, over-expression of the carrier in procyclic forms increased parasite susceptibility more than 13-fold. Metabolomic analyses of parasites over-expressing TbMCP14 showed increased levels of the proline metabolite, pyrroline-5-carboxylate, suggesting a possible involvement of TbMCP14 in energy production. The generation of TbMCP14 knock-out parasites showed that the carrier is not essential for survival of *T*. *brucei* bloodstream forms, but reduced parasite proliferation under standard culture conditions. In contrast, depletion of TbMCP14 in procyclic forms resulted in growth arrest, followed by parasite death. The time point at which parasite proliferation stopped was dependent on the major energy source, i.e. glucose versus proline, in the culture medium. Together with our findings that proline-dependent ATP production in crude mitochondria from TbMCP14-depleted trypanosomes was reduced compared to control mitochondria, the study demonstrates that TbMCP14 is involved in energy production in *T*. *brucei*. Since TbMCP14 belongs to a trypanosomatid-specific clade of mitochondrial carrier family proteins showing very poor similarity to mitochondrial carriers of mammals, it may represent an interesting target for drug action or targeting.

## Author Summary

Human and animal trypanosomiases caused by *Trypanosoma brucei* parasites represent major burdens to human welfare and agricultural development in rural sub-Saharan Africa. Although the numbers of infected humans have decreased continuously during the last decades, emerging resistance and adverse side effects against commonly used drugs require an urgent need for the identification of novel drug targets and the development of new drugs. Using an unbiased genome-wide screen to search for genes involved in the mode of action of trypanocidal compounds, we identified a member of the mitochondrial carrier family, TbMCP14, as prime candidate to mediate the action of a group of anti-parasitic choline analogs against *T*. *brucei*. Ablation of TbMCP14 expression by RNA interference or gene deletion decreases the susceptibility of parasites towards the compounds while over-expression of the carrier shows the opposite effect. In addition, down-regulation of TbMCP14 protects mitochondria from drug-induced decrease in mitochondrial membrane potential and reduces proline-dependent ATP production. Together, the results demonstrate that TbMCP14 is involved in energy production in *T*. *brucei*, possibly by acting as a mitochondrial proline carrier, and reveal TbMCP14 as candidate protein for drug action or targeting.

## Introduction

African trypanosomes are vector-borne protozoans that cause serious public health problems and severe economic losses in sub-Saharan African countries. The current chemotherapy against *Trypanosoma brucei*, the causative agent of human African trypanosomiasis, or sleeping sickness, and the related cattle disease Nagana, is based on multiple injections of drugs some of which are associated with serious side effects. Treatment of Nagana is commonly based on mutagenic drugs including ethidium bromide and isometamidium chloride, and also on suramin and diminazene aceturate, both with considerable toxicity to cattle [1]. In the case of human African trypanosomiasis, pentamidine and suramin are widely used during the first stage of the disease, when the parasites are confined to the hemolymphatic system. The second stage of sleeping sickness, which is characterized by parasite invasion of the central nervous system, is treated with melarsoprol or a combination of nifurtimox/eflornithine [2]. Melarsoprol, the only drug against both forms of sleeping sickness, which are caused by *T*. *b*. *gambiense* or *T*. *b*. *rhodesiense*, is highly toxic causing encephalopathies in 5% of cases. In addition, there are well-known examples of drug resistance in the field [3,4]. In the laboratory, trypanosome drug resistance has frequently been found to involve loss of nutrient transporters: the aminopurine transporter TbAT1 for melaminophenyl arsenicals and diamidines [5], the aquaglyceroporin TbAQP2 for melarsoprol and pentamidine [6], and the amino acid permease TbAAT6 for eflornithine [7–9]. These transporters (i) import drugs in addition to their natural substrates [7,10–12] and (ii) are not essential [6,7,13]. Loss-of-function mutations in the corresponding genes can therefore render the trypanosomes resistant by reducing drug uptake. However, a number of trypanocides accumulate in the trypanosomes' single mitochondrion, possibly targeting mitochondrial structures including the kinetoplast [14–16], the intercatenated network of circular DNA molecules that comprises the parasite’s mitochondrial genome. This implies that transporters of the (inner) mitochondrial membrane are also involved in drug accumulation and activity. The nature of these transporters, and whether they play a role in drug resistance, is unknown.

*T*. *brucei* and other protozoan parasites acquire nutrients and building blocks of macromolecules for rapid cell proliferation from their mammalian or insect hosts. However, recent reports have shown that trypanosomatids not only acquire lipids for membrane formation from the environment, but are also capable of *de novo* synthesis of all major membrane lipid classes (reviewed in [17]). The most abundant phospholipid class in *T*. *brucei* is phosphatidylcholine (PC) [18], which can be generated by acylation of lyso-PC taken up from the host [19]. Alternatively, PC can be produced from host-derived choline [20] by sequential action of three enzymes via the CDP-choline pathway [17]. This pathway is essential for survival of *T*. *brucei* parasites in culture [17].

PC is also the most abundant phospholipid class in malaria parasites (*Plasmodium spp*.)(reviewed in [21]). Its synthesis can occur via multiple routes, including the CDP-choline pathway (reviewed in [22,23]). The inability to knock out individual genes involved in this pathway suggests that PC formation via CDP-choline is essential in *Plasmodium* [24]. In addition, uptake of the substrate for this pathway, choline, can be inhibited by a set of choline analogs, which have been found to be toxic for malaria parasites, at nanomolar concentrations [25–27]. Although the primary target of the drugs is likely the inhibition of choline uptake, resulting in inhibition of PC synthesis [28–30], other mechanisms of action have been proposed [31,32]. Structural refinements of the drugs has led to the development of third- and fourth-generation compounds, one of which, named T3 (or albitiazolium), is currently in clinical trials to treat severe malaria [30]. More recently, a subset of these compounds has also been shown to be toxic for *T*. *brucei* and Leishmania parasites at (sub-) micromolar concentrations [33]. Their mode of action is, however, unclear: although they effectively inhibit choline uptake and, thus, de novo PC formation in *T*. *brucei* [20], they may kill trypanosomes by affecting mitochondrial structure and function [20,33].

In the present study, we used three of the leading choline analogs, a bis-quartenary ammonium salt, G25 [34], and two bis-tiazolium salts, T3 and T4 [35], to elucidate their site(s) and mode(s) of action against *T*. *brucei*. For this, we screened an RNAi library previously established in *T*. *brucei* bloodstream forms [9] to identify genes conferring parasite resistance towards the choline analogs. Interestingly, we found that treatment of *T*. *brucei* bloodstream forms with these drugs selected parasite populations in which the expression of a gene encoding a member of the mitochondrial carrier protein family, MCP14, was down-regulated. Expression of MCP14 was found to be essential for normal growth of both bloodstream and procyclic form trypanosomes in culture.

## Results

### Screening of RNAi library and identification of TbMCP14

**Fig 1:**
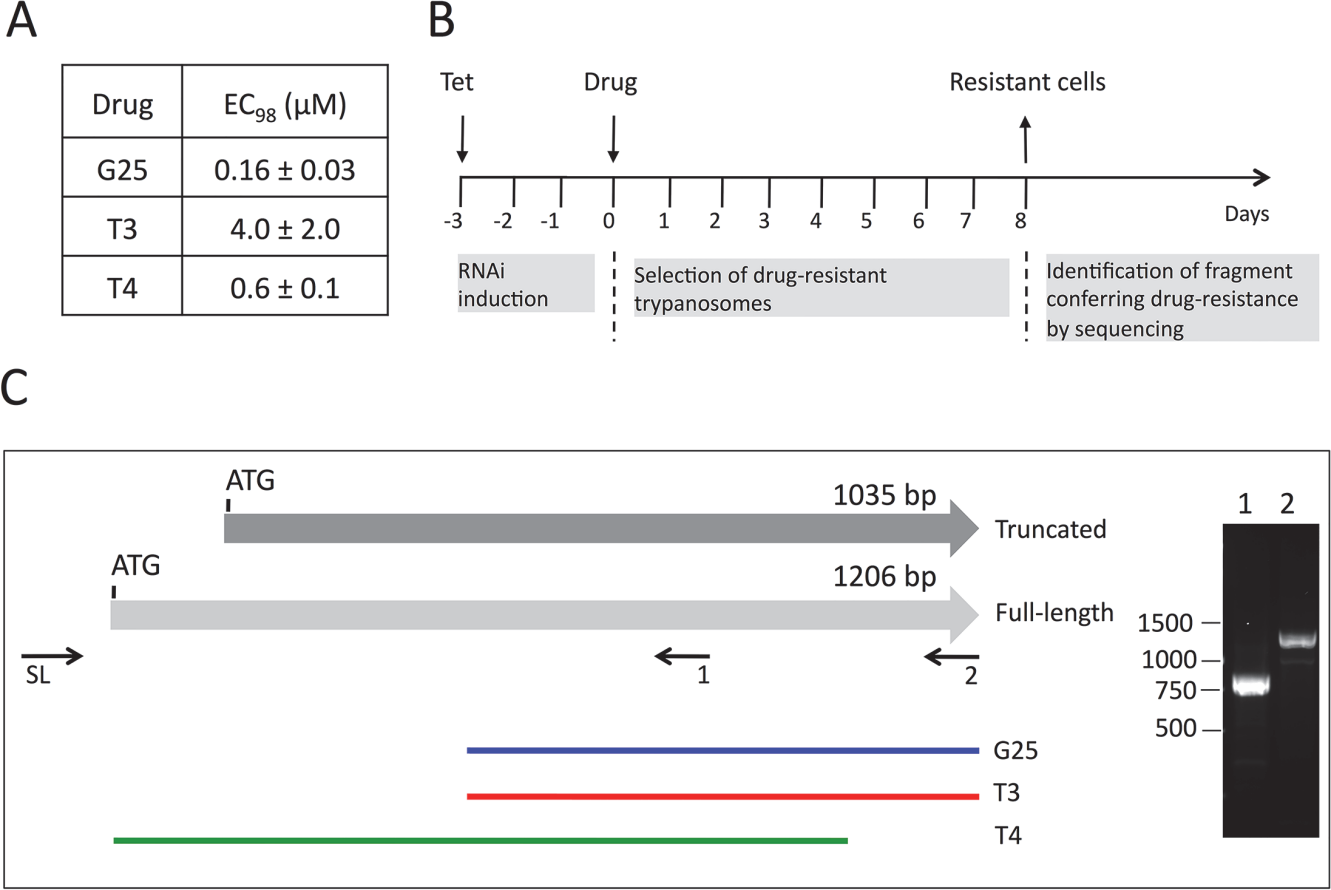
Screening of RNAi library and identification of TbMCP14. A) The concentrations of drugs killing 98% (EC_98_) of *T*. *brucei* bloodstream forms were determined using Alamar blue assays. The numbers represent mean values ± standard deviations from 3 independent experiments. B) Time-line of induction of RNAi library, selection of resistant clones, and sequencing of RNAi inserts from resistant parasites. C) Scheme depicting truncated and full-length TbMCP14. The horizontal grey arrows represent two potential TbMCP14 ORFs, encoding proteins of 344 (truncated) and 401 amino acids (full-length). The colored lines at the bottom indicate the alignments of the RNAi inserts isolated from resistant parasites after selection with G25, T3 and T4 (from top to bottom). The right panel shows an ethidium bromide-stained agarose gel with the RT-PCR products amplified using a primer corresponding to the spliced leader sequence (SL) together with the gene-specific primers 1 and 2 indicated below full-length TbMCP14. The numbers on the left indicate the migration of base pair markers. TbMCP14 was the only gene identified in the RNAi screens using either of the three drugs.

Recently, an inducible RNAi library has been established in *T*. *brucei* bloodstream forms, which allows an unbiased approach to identify genes involved in drug uptake or action [9]. We have used this library to elucidate the mode and site of action of a set of choline analogs known to be toxic for parasitic protozoa, including *T*. *brucei* [27,33]. In a first step, we determined the concentrations of G25, T3 and T4 required to kill 98% of *T*. *brucei* bloodstream forms (EC_98_) after 3 days of culture using Alamar blue assays [9] (Fig 1A). These concentrations were subsequently used to treat separate trypanosome cultures with G25, T3 and T4, following induction of RNAi for 60 h with tetracycline (Fig 1B). After 8 days, parasites cultured in the absence of tetracycline were dead, while resistant trypanosomes started to proliferate in cultures incubated with tetracycline. After another 3 days of culture, during which time parasites were kept in fresh medium to allow optimal growth, DNA was extracted and inserts potentially conferring resistance towards the drugs were amplified using specific primers [9]. Interestingly, the individual screens using G25, T3 and T4 all resulted in selection of trypanosomes bearing RNAi inserts partially covering the gene encoding putative mitochondrial carrier protein 14 (TbMCP14; Tb927.10.13120), a member of a large family of mitochondrial carriers [36]. Screening with G25 and T3 selected parasites bearing the same RNAi insert, while T4 selected parasites harboring a different TbMCP14 RNAi sequence (Fig 1C, green line). Tb927.10.13120 was annotated to comprise an ORF of 1035 bp (TriTrypDB, GeneDB), encoding a protein of 344 amino acids. A C-terminally tagged product of this ORF was shown to localize to the mitochondrion [36]. However, recent results from transcriptome analyses suggested that a second potential start codon 171 bp upstream of the annotated ATG might exist [37,38]. RT-PCR with a spliced leader primer (primer 12, S1 Table) and TbMCP14 reverse primers (primers 2 and 4, S1 Table) generated products consistent with Tb927.10.13120 encoding an mRNA of 1206 bp (Fig 1C, right panel), resulting in a predicted full-length protein of 401 amino acids.

### TbMCP14 is closely linked to drug action

**Fig 2:**
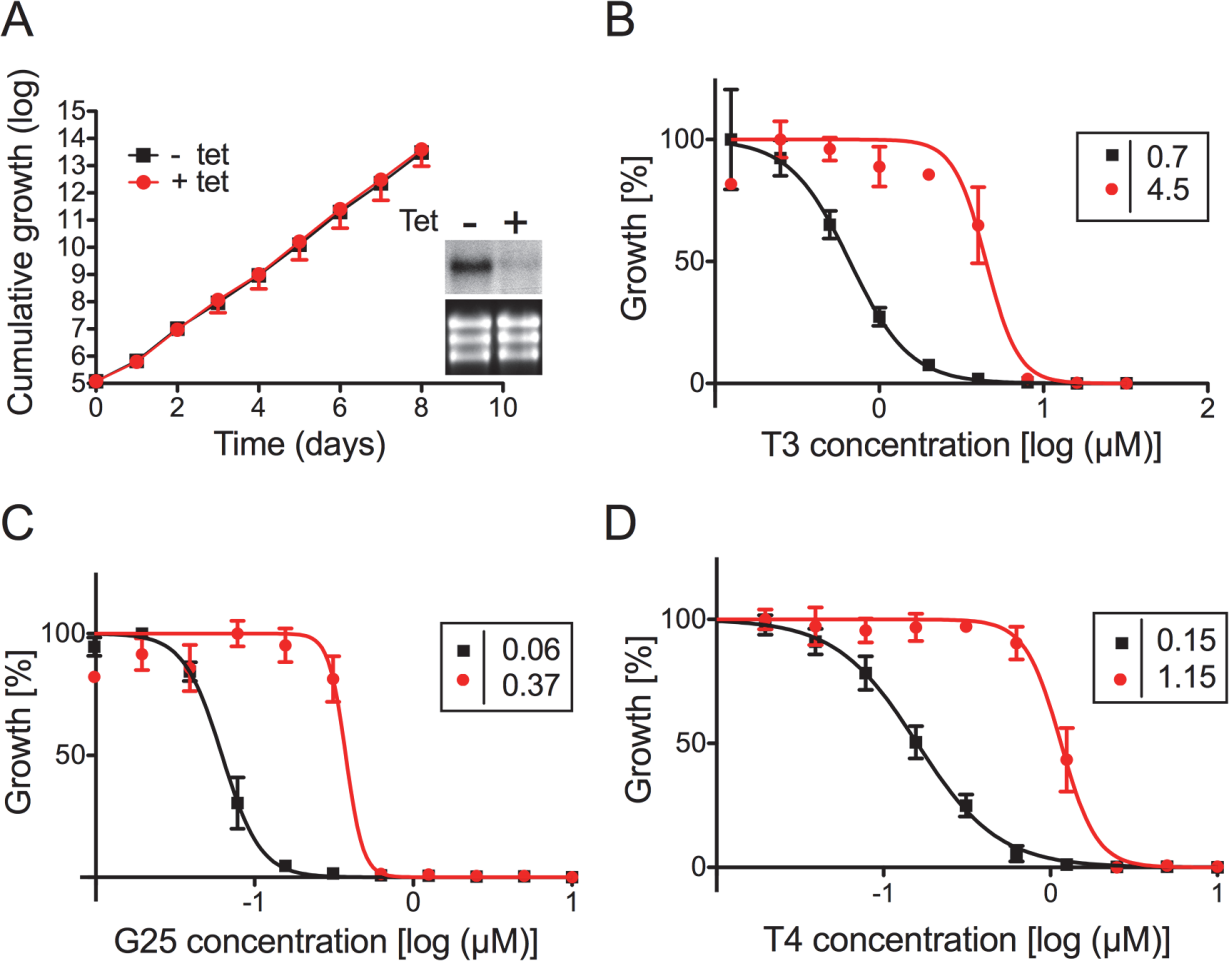
Sensitivity of *T*. *brucei* bloodstream forms towards choline analogs after RNAi against TbMCP14. A) Growth curves of RNAi parasites cultured in the absence (black squares,-tet) or presence (red circles, +tet) of tetracycline to induce RNAi against TbMCP14. Data points represent mean values ± standard deviations from three independent experiments. The inset shows a Northern blot analysis of TbMCP14 mRNA levels from parasites incubated for 3 days in the absence (-) or presence (+) of tetracycline (upper panels); rRNA levels are shown as loading control (lower panels). B,C,D) Alamar blue assays to determine the sensitivities of RNAi parasites towards T3 (panel B), G25 (panel C) and T4 (panel D) after down-regulation of TbMCP14 expression. Squares and circles represent trypanosomes cultured in the absence and presence, respectively, of tetracycline (added three days before the assay). The data points represent mean values ± SEM of triplicate determinations from single experiments. The insets show the EC_50_ values of the respective curves.

**Fig 3:**
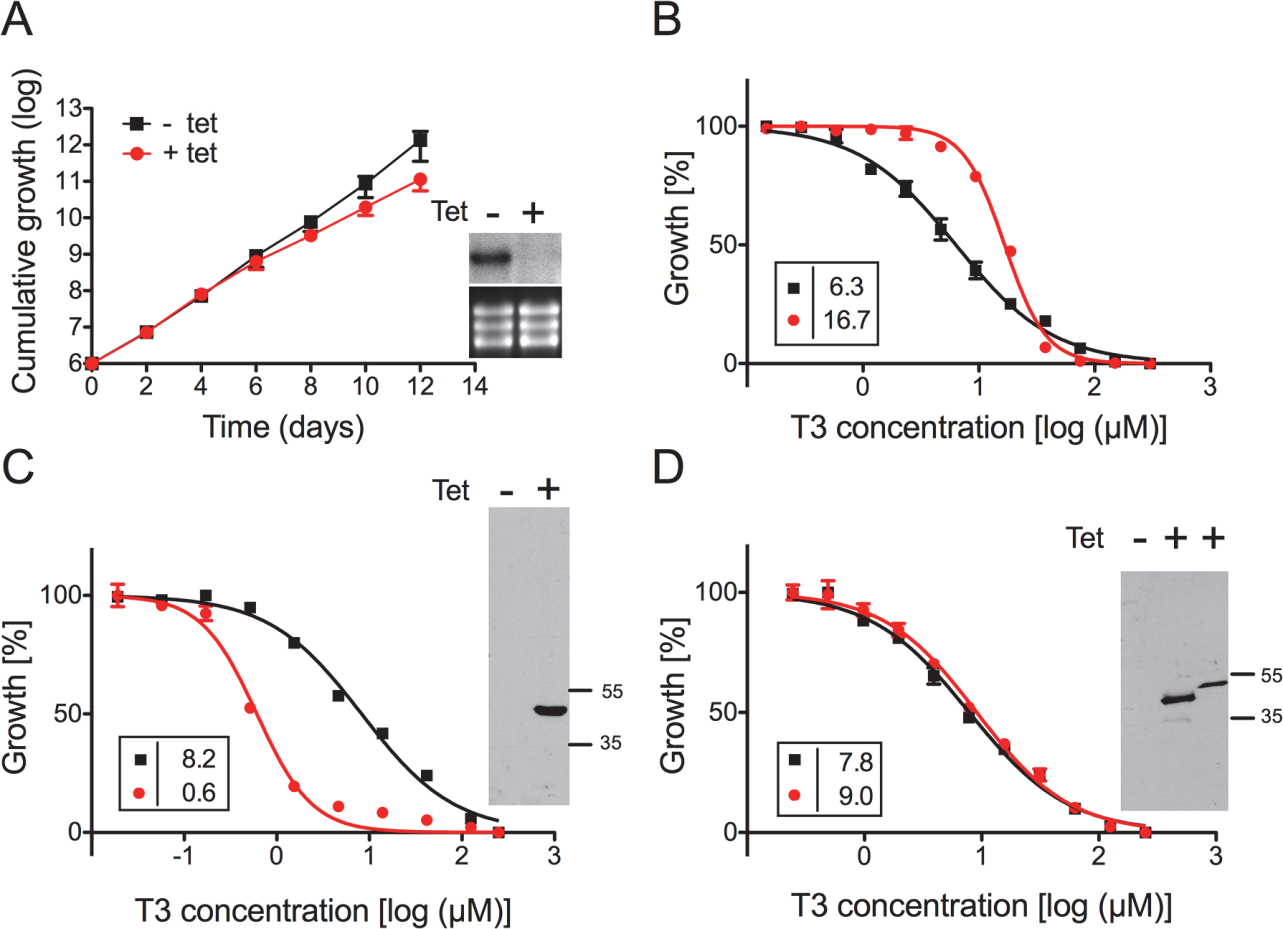
Sensitivity of *T*. *brucei* procyclic forms towards T3 after modulation of TbMCP14 expression. A) Growth curves of RNAi parasites cultured in the absence (black squares,-tet) or presence (red circles, +tet) of tetracycline to induce RNAi against TbMCP14. Data points represent mean values ± standard deviations from three independent experiments. The inset shows a Northern blot analysis of TbMCP14 mRNA levels from parasites incubated for 3 days in the absence (-) or presence (+) of tetracycline (upper panels); rRNA levels are shown as loading control (lower panels). B) Alamar blue assays to determine the sensitivity of parasites towards T3 after RNAi-mediated down-regulation of TbMCP14 (panel B), or over-expression of full-length (panel C) or truncated (panel D) TbMCP14. Black squares and red circles represent trypanosomes cultured in the absence and presence, respectively, of tetracycline. The data points represent mean values ± SEM of triplicate determinations from a typical experiment. The insets on the left show the EC_50_ values of the respective curves. The insets on the right in panels C and D represent SDS-PAGE/immunoblot analyses using anti-cMyc antibody on protein lysates from parasites cultured in the absence (-) or presence (+) of tetracycline to induce over-expression of full-length (panel C, right lane) or truncated (panel D, middle lane) TbMCP14; the right lane in the inset of panel D represents full-length TbMCP14 for size comparison with truncated TbMCP14.

To verify the involvement of TbMCP14 in drug resistance towards choline analogs, expression of Tb927.10.13120 was down-regulated by inducible RNAi in *T*. *brucei* bloodstream forms. Addition of tetracycline to parasites in culture showed no growth phenotype, despite efficient reduction in Tb927.10.13120 transcript level (Fig 2A). The non-essentiality of Tb927.10.13120 as assessed by RNAi was not surprising since trypanosomes selected from the RNAi library were viable after 11 days of RNAi induction (see above). However, treatment of parasites after down-regulation of Tb927.10.13120 expression showed increased resistance towards G25, T3, and T4 (Fig 2B–2D). In Alamar blue assays, the EC_50_ values of TbMCP14-depleted bloodstream form parasites for the compounds increased an average of 7-fold compared to uninduced cells. Subsequently, Tb927.10.13120 expression was also down-regulated using RNAi in *T*. *brucei* procyclic forms. Incubation of parasites in the presence of tetracycline showed disappearance of Tb927.10.13120 mRNA levels and a small growth defect (Fig 3A). In line with the results obtained for bloodstream forms (Fig 2B–2D), RNAi against Tb927.10.13120 conferred increased resistance (approximately 3-fold) of procyclic form parasites towards T3 (Fig 3B).

To further confirm the involvement of TbMCP14 in drug action, we over-expressed a tetracycline-inducible ectopic copy of cMyc-tagged Tb927.10.13120 in procyclic and bloodstream form trypanosomes. Analysis by SDS-PAGE and immunoblotting showed that cMyc-TbMCP14 was expressed in the presence, but not in the absence, of tetracycline as an approximately 48 kDa protein, and that expression of full-length cMyc-TbMCP14 conferred increased susceptibility (>13-fold) to parasites against T3 (Fig 3C). These results clearly demonstrate that the tagged version of the full-length protein is functional. In contrast, no effect on sensitivity towards T3 was observed in parasites over-expressing a tagged version of the short (truncated) form of TbMCP14, which migrated with an apparent molecular mass of 42 kDa (Fig 3D).

In control experiments, drug sensitivity was assessed in *T*. *brucei* procyclic forms over-expressing another member of the MCP family, TbMCP5. This protein has previously been shown to act as ADP/ATP carrier [39]. In contrast to procyclic form trypanosomes over-expressing TbMCP14 (Fig 3C), expression of N-terminally cMyc-tagged TbMCP5 (which is known to be functional [39]) had no major effect on the EC_50_ value for T3 (S1A and S1B Fig).

Additionally, viability of *T*. *brucei* bloodstream forms over-expressing cMyc-TbMCP14 (full-length) cultured in the presence of various concentrations of G25 was investigated by propidium iodide (PI) staining. While dead parasites are permeable to PI, resulting in its intercalation into DNA with subsequent fluorescence emission (PI positive), living parasites are impermeable to PI and show no emission of fluorescence. The results show that over-expression of TbMCP14 slightly increased toxicity of G25 (S2 Fig, top panels), whereas its down-regulation had a protective effect on parasite survival in the presence of the drug (S2 Fig, bottom panels). In addition, the data show that G25 is only toxic for *T*. *brucei* bloodstream forms after prolonged incubation, i.e. after 72 h (S2 Fig, top left, green line). Taken together, the results demonstrate that TbMCP14 expression is closely linked to the action of the choline analogs, G25, T3 and T4.

### Localization of TbMCP14

**Fig 4:**
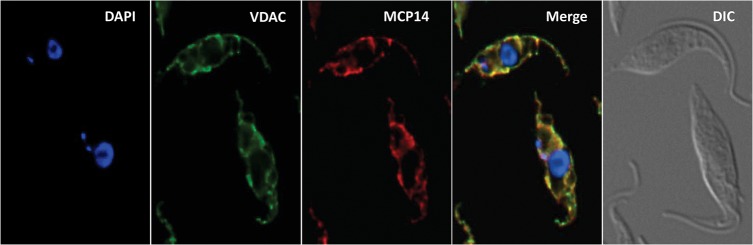
Localization of TbMCP14. *T*. *brucei* procyclic forms expressing cMyc-tagged TbMCP14 were fixed and stained for DNA with DAPI, anti-VDAC antibody as mitochondrial marker, and anti-cMyc to reveal TbMCP14. The merge shows mitochondrial co-localization of TbMCP14 and VDAC. Trypanosomes are shown by differential interference contrast (DIC) optics.

In a previous report, the truncated version of TbMCP14 was expressed as cMyc-tagged version that was found to localize in the mitochondrion [36]. We now show by immunofluorescence microscopy that also full-length TbMCP14 localizes to the mitochondrion, co-localizing with the mitochondrial marker protein voltage-dependent anion channel (VDAC) (Fig 4). The results further demonstrate that the N-terminal 57 amino acids of full-length TbMCP14 are not essential for correct targeting of the protein to the mitochondrion, although they are required for drug susceptibility.

### TbMCP14 mediates the effects of choline analogs on mitochondrial membrane potential

**Fig 5:**
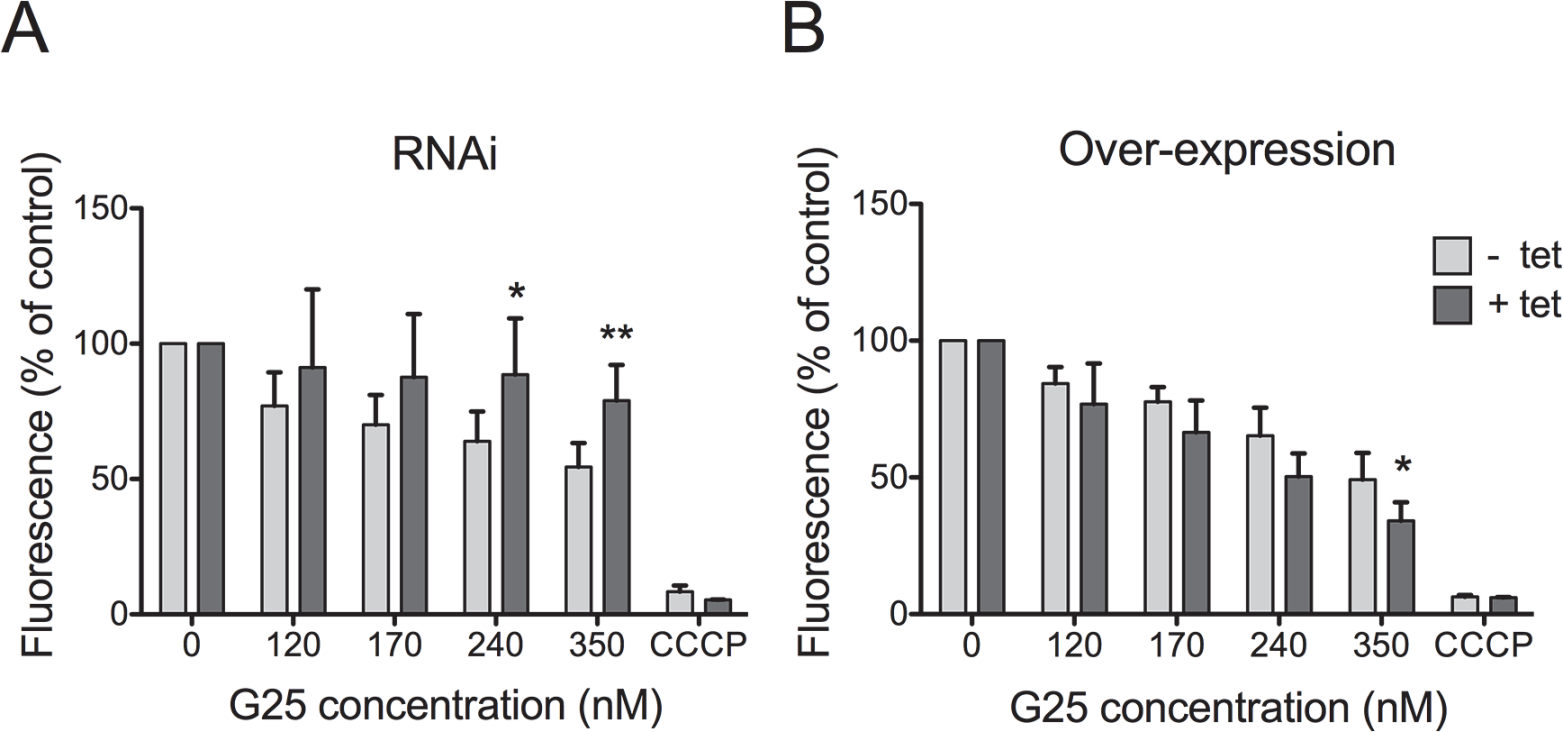
Analysis of mitochondrial membrane potential (ΔΨ_m_). *T*. *brucei* bloodstream forms were incubated in the absence (light grey bars) or presence (dark grey bars) of tetracycline to down-regulate TbMCP14 by RNAi (panel A) or over-express TbMCP14 (panel B) for 3 days before treatment with G25. Parasites were treated with different concentrations of G25 for 24 h followed by addition of 25 nM TMRE for an additional 30 min and analysis by flow cytometry. Carbonyl cyanide *m*-chlorophenyl hydrazone (CCCP) was added to control cultures to disrupt ΔΨ_m_. The values were normalized relative to untreated control trypanosomes. The data points represent mean values ± SD from at least four independent experiments. The asterisks represent statistical difference relative to the respective uninduced control (*p<0.05, **p<0.01, unpaired student’s t test).

It has been suggested that the trypanocidal activity of choline analogs may occur via modulation of mitochondrial membrane potential (ΔΨ_m_) [33]. We revisited this proposal by measuring drug-induced changes in ΔΨ_m_ in *T*. *brucei* bloodstream forms, in which TbMCP14 was over-expressed or down-regulated. Using the mitochondrial dye tetramethylrhodamine ethyl ester (TMRE) and flow cytometry, we detected a decrease in ΔΨ_m_ following treatment of parasites for 24 h with increasing concentrations of G25 (Fig 5A). This drug-induced decrease in ΔΨ_m_ was largely prevented in parasites after RNAi-mediated down-regulation of TbMCP14. Conversely, sensitivity of ΔΨ_m_ to high concentrations of G25 was slightly increased in parasites after over-expression of TbMCP14 (Fig 5B). In control experiments, parasite viability during drug treatment was measured by incorporation of PI and flow cytometry analysis. We found no difference in PI staining between parasites before and after incubation for 24 h in the presence of G25, ruling out the possibility that the observed decrease in ΔΨ_m_ was the result of parasite death, rather than drug action (S3 Fig). Together, these results demonstrate that TbMCP14 is involved in drug-induced alterations of ΔΨ_m_, which clearly is an early event during G25 action.

### TbMCP14 is involved in the mode of action of pentamidine

**Fig 6:**
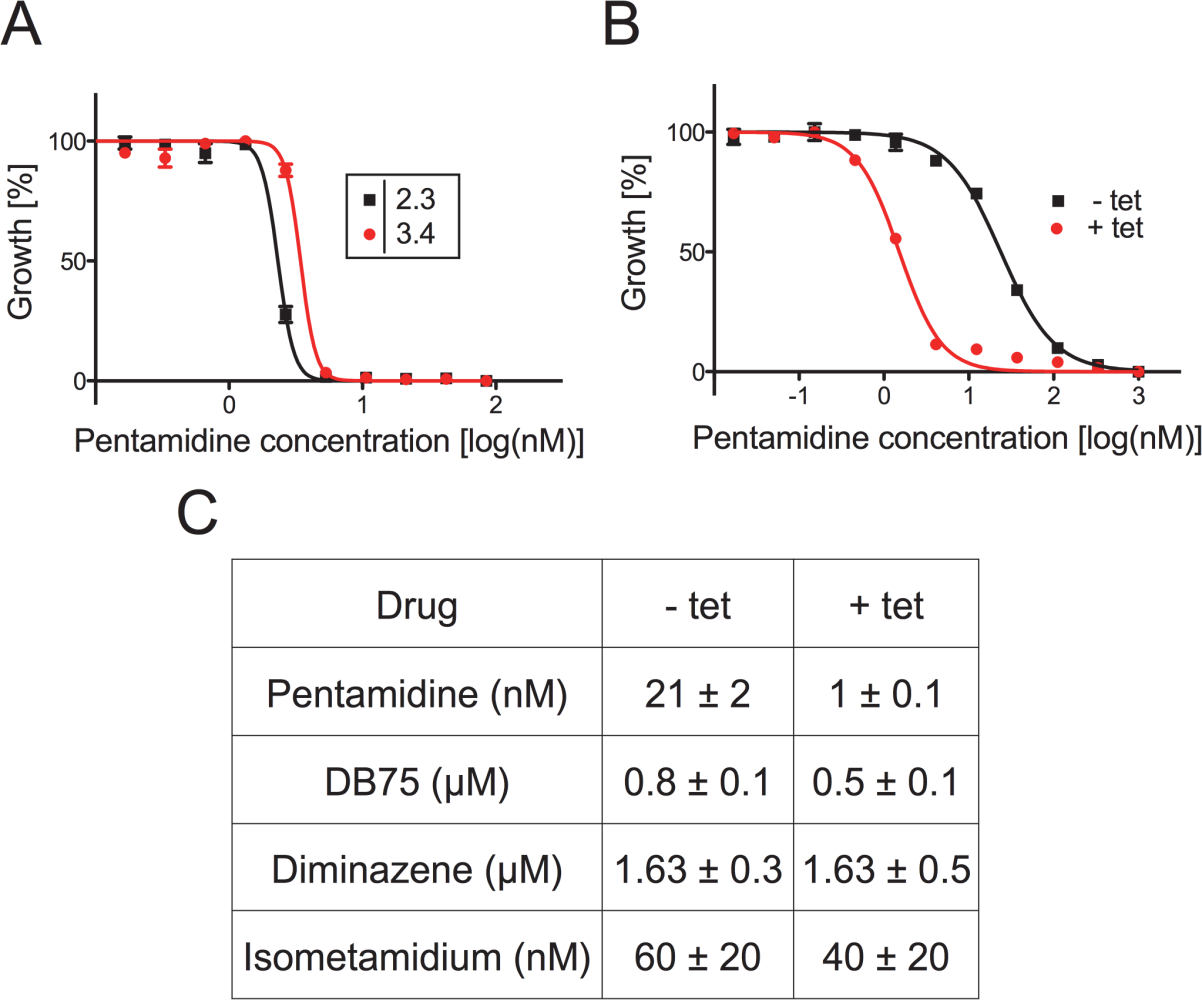
Sensitivity of *T*. *brucei* towards trypanocidal cations. A) Sensitivity to pentamidine of bloodstream forms cultured in the absence (black squares) or presence (red circles) of tetracycline to down-regulate TbMCP14 expression. Data points are from one of two experiments and represent mean values ± SEM of triplicate determinations. B) Sensitivity to pentamidine of procyclic forms cultured in the absence (black squares) or presence (red circles) of tetracycline to induce TbMCP14 over-expression. Data points are from one of three experiments and represent mean values ± SEM of triplicate determinations. C) Compilation of EC_50_ values for different trypanocidal cations for procyclic forms cultured in the absence (-tet) or presence (+tet) of tetracycline to over-express TbMCP14. The numbers represent mean values ± standard deviations from three independent experiments.

Although the mode of action of diamidines is likely multi-factorial, there is evidence that pentamidine and other cationic trypanocides may accumulate inside mitochondria and cause a decrease of ΔΨ_m_ in kinetoplastids [15,40–42]. For this reason we studied if TbMCP14 might be involved in the mode of action of diamidines and the quaternary ammonium phenanthridine isometamidium by assessing the sensitivity of trypanosomes after over-expression or down-regulation of TbMCP14 to three diamidines, pentamidine, DB75 and diminazene aceturate, as well as isometamidium chloride (Fig 6). Interestingly, we found that over-expression of TbMCP14 in procyclic forms resulted in an approximately 14-fold increase in parasite susceptibility towards pentamidine (Fig 6). However, only small changes in pentamidine sensitivity were seen after depletion of TbMCP14. It is possible that the remaining levels of TbMCP14 (see above) masked possible effects of these compounds on RNAi parasites, or that the primary targets of pentamidine are cytosolic, or in other sub-cellular compartments. In contrast to pentamidine, no significant changes in toxicity were found for DB75, diminazene aceturate, or isometamidium chloride upon over-expression of TbMCP14 (Fig 6C). In addition, sensitivity of procyclic form trypanosomes towards pentamidine was unaffected in parasites over-expressing TbMCP5 (S1C Fig).

### Generation of TbMCP14 knock-out parasites

**Fig 7:**
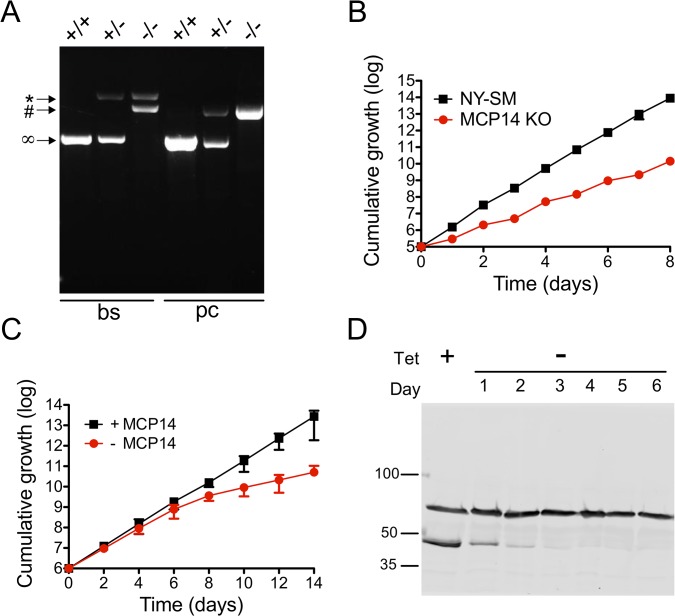
Analysis and growth of *T*. *brucei* bloodstream and procyclic form TbMCP14 (conditional) null mutants. A) Agarose gel of PCR products obtained using primers binding in the UTR regions outside the TbMCP14 ORF. In bloodstream form (bs) parasites, the two endogenous TbMCP14 alleles (∞, 1363 bp) were replaced by hygromycin (*, 2762 bp) and blasticidin (#, 2135 bp) resistance genes, whereas in procyclic forms (pc), the endogenous alleles were replaced by blasticidin (#, 2135 bp) and phleomycin (#, 2111 bp) resistance genes. The numbers on the right indicate the migration of base pair markers. B) Growth of *T*. *brucei* bloodstream form TbMCP14 null mutants in culture (red circles), in comparison with the parental cell line (black squares, indicated as NY-SM). C) Growth of *T*. *brucei* procyclic form TbMCP14 conditional null mutants in the presence (black squares, +MCP14) or absence (red circles,-MCP14) of tetracycline. Data points in B and C represent mean values ± standard deviations from three independent experiments; for some data points, the error bars are smaller than the symbols. D) SDS-PAGE/immunoblot analysis of lysates from *T*. *brucei* procyclic form TbMCP14 conditional null mutants grown in the presence (+) or absence (-) of tetracycline for indicated times. cMyc-tagged TbMCP14 was visualized with anti-cMyc antibody. Hsp70 was visualized with anti-HSP70 antibody and represents a loading control. Molecular mass markers are indicated.

To further study the importance of TbMCP14 in *T*. *brucei* viability, we generated bloodstream and procyclic form Tb927.10.13120 (conditional) null mutants. Since RNAi against TbMCP14 in bloodstream forms showed no growth defect, we attempted to generate straight knock-out parasites by sequentially deleting the two endogenous Tb927.10.13120 alleles. Successful replacement of the first and second alleles by blasticidin resistance and phleomycin resistance genes, respectively, was verified by PCR (Fig 7A). The resulting TbMCP14 null bloodstream forms were viable, but they showed reduced growth in culture compared to the parental cell line (Fig 7B). The cell doubling time of the null mutant was calculated to be 10.0 ± 0.8 h, compared to 6.3 ± 0.1 h (mean values ± standard deviations from three independent experiments) of the parental strain. Together, the results show that TbMCP14 is essential for normal growth of *T*. *brucei* bloodstream forms in culture, but non-essential for viability under these conditions.

Because *T*. *brucei* procyclic forms showed slightly reduced growth in culture after RNAi against TbMCP14 (Fig 3A), we followed a different strategy to obtain procyclic form TbMCP14 conditional null mutants by introducing a tetracycline-inducible ectopic copy of Tb927.10.13120 before knocking out the second allele. To be able to monitor expression of ectopic TbMCP14, the gene was extended with a sequence encoding cMyc at its 3’ end. Again, successful deletion of the two endogenous alleles was verified by PCR (Fig 7A). Resulting parasites cultured in the presence of tetracycline, i.e. expressing cMyc-tagged ectopic TbMCP14, grew normally in standard growth medium (Fig 7C). In contrast, after removal of tetracycline from the culture medium, parasites showed reduced growth after 6 days of culture (Fig 7C). SDS-PAGE and immunoblotting revealed that cMyc-tagged TbMCP14 was expressed in parasites cultured in the presence of tetracycline, but disappeared during ablation of TbMCP14 expression after removal of tetracycline (Fig 7D). Together, the results show that TbMCP14 is essential for growth of *T*. *brucei* procyclic forms in culture and confirms that cMyc-tagged TbMCP14 is functional (see also Fig 3C).

### TbMCP14 is involved in energy metabolism

**Fig 8:**
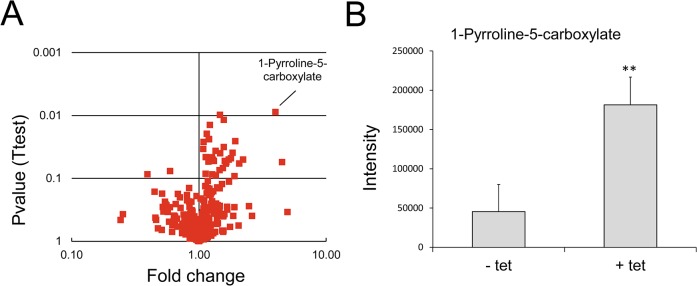
Analysis of metabolites after over-expression of TbMCP14 in procyclic forms. A) A volcano plot of metabolites identified in cells over-expressing TbMCP14 reveals that a metabolite with a mass consistent with it being pyrroline-5-carboxylate was the only metabolite showing a greater than 3-fold change in abundance with statistical significance (p<0.05). B) Pyrroline-5-carboxylate intensities in lysates from parasites cultured in the presence (+ tet) or absence (- tet) of tetracycline to over-express TbMCP14. The asterisks represent statistical difference relative to the respective uninduced controls (**p<0.01, unpaired student’s t test).

**Fig 9:**
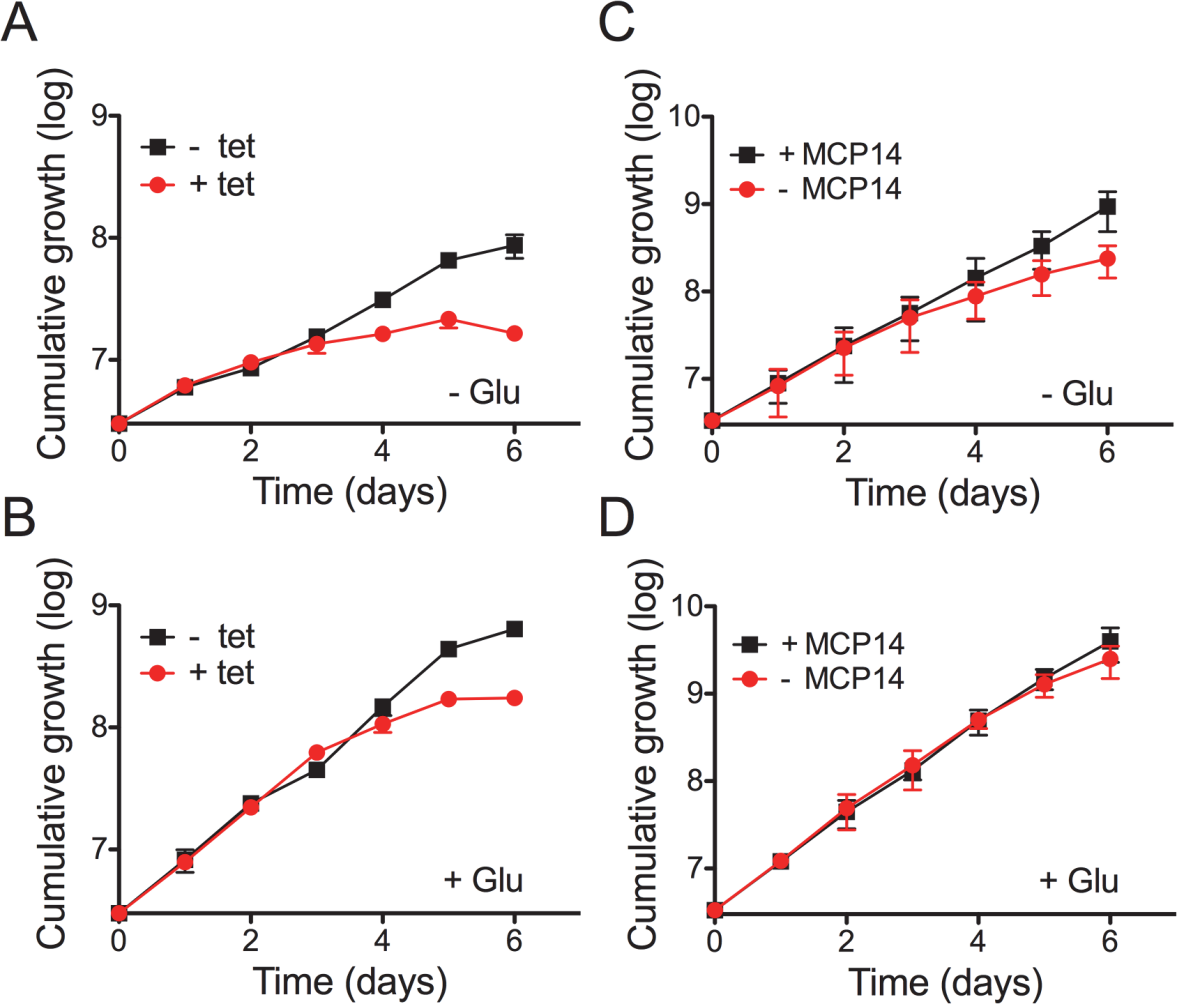
Effect of knock-down of TbMCP14 on growth of *T*. *brucei* procyclic forms cultured in glucose-depleted medium. A) Parasites were grown in SDM80 containing normal (5.5 mM; panel A) or low (0.15 mM; panel B) levels of glucose in the absence (black squares) or presence (red circles) of tetracycline to induce RNAi-mediated down-regulation of TbMCP14. C,D) TbMCP14 conditional knock-out parasites were grown in glucose-depleted (0.15 mM, panel C) or glucose-containing SDM80 (5.5 mM, panel D) in the presence (black squares, +MCP14) or absence (red circles,-MCP14) of tetracycline to maintain or deplete, respectively, TbMCP14. All data points represent mean values ± standard deviations from three independent experiments; for some data points, the error bars are smaller than the symbols.

To elucidate the physiological function of TbMCP14, we performed untargeted metabolomic analyses of small metabolites [43] in *T*. *brucei* procyclic forms after over-expression of TbMCP14. This approach revealed that a metabolite with a mass compatible with pyrroline-5-carboxylate, was the only metabolite showing a greater than 3-fold change in abundance with statistical significance (P<0.05) in parasites over-expressing TbMCP14 (Fig 8). Pyrroline-5-carboxylate is the degradation product of proline produced in the mitochondrion by the action of proline dehydrogenase [44], suggesting that TbMCP14 might play a role in proline metabolism. Since proline is abundant in the culture medium, changes in this amino acid are not apparent, nor are changes to glutamate, the amino acid formed from pyrroline-5-carboxylate whose mitochondrial abundance is negligible compared to its abundance in the culture medium. Glutamate is then converted to 2-ketoglutarate, which is also produced from glucose, as are other carboxylic acids such as succinate, fumarate and malate, which explains why pyrroline-5-carboxylate is the only discriminatory metabolite identified in these experiments. Based on this finding, we investigated if down-regulation of TbMCP14 may show a more pronounced growth defect if procyclic form trypanosomes were cultured in glucose-depleted medium (SDM80), in which they are known to increase consumption of amino acids, in particular proline, for energy production by >6 times compared to parasites grown in standard medium containing glucose (SDM79) [44]. Our results showed that after RNAi against TbMCP14, trypanosomes showed a much stronger growth defect in SDM80 compared to SDM79, resulting in growth arrest after 5 days of induction (Fig 9A, compare with Fig 3A). When SDM80 was supplemented with 5.5 mM glucose, the growth defect was delayed (Fig 9B), demonstrating that the availability of glucose improved parasite growth in TbMCP14-depleted cells. Together, these results indicate that TbMCP14 is likely involved in metabolism of proline for energy production. In line with this interpretation, we found that depletion of TbMCP14 in conditional knock-out parasites cultured in glucose-depleted medium (SDM80) again reduced parasite growth (Fig 9C) compared to parasites grown in the presence of glucose (Fig 9D). In contrast, depletion of TbMCP14 had no effect on growth of trypanosomes cultured in glucose-supplemented SDM80 for up to 6 days (Fig 9C). Culturing conditional knock-out parasites in glucose-depleted medium had no effect on ablation of TbMCP14 expression (S4 Fig).

### Depletion of TbMCP14 affects proline-dependent ATP production in crude mitochondria

**Fig 10:**
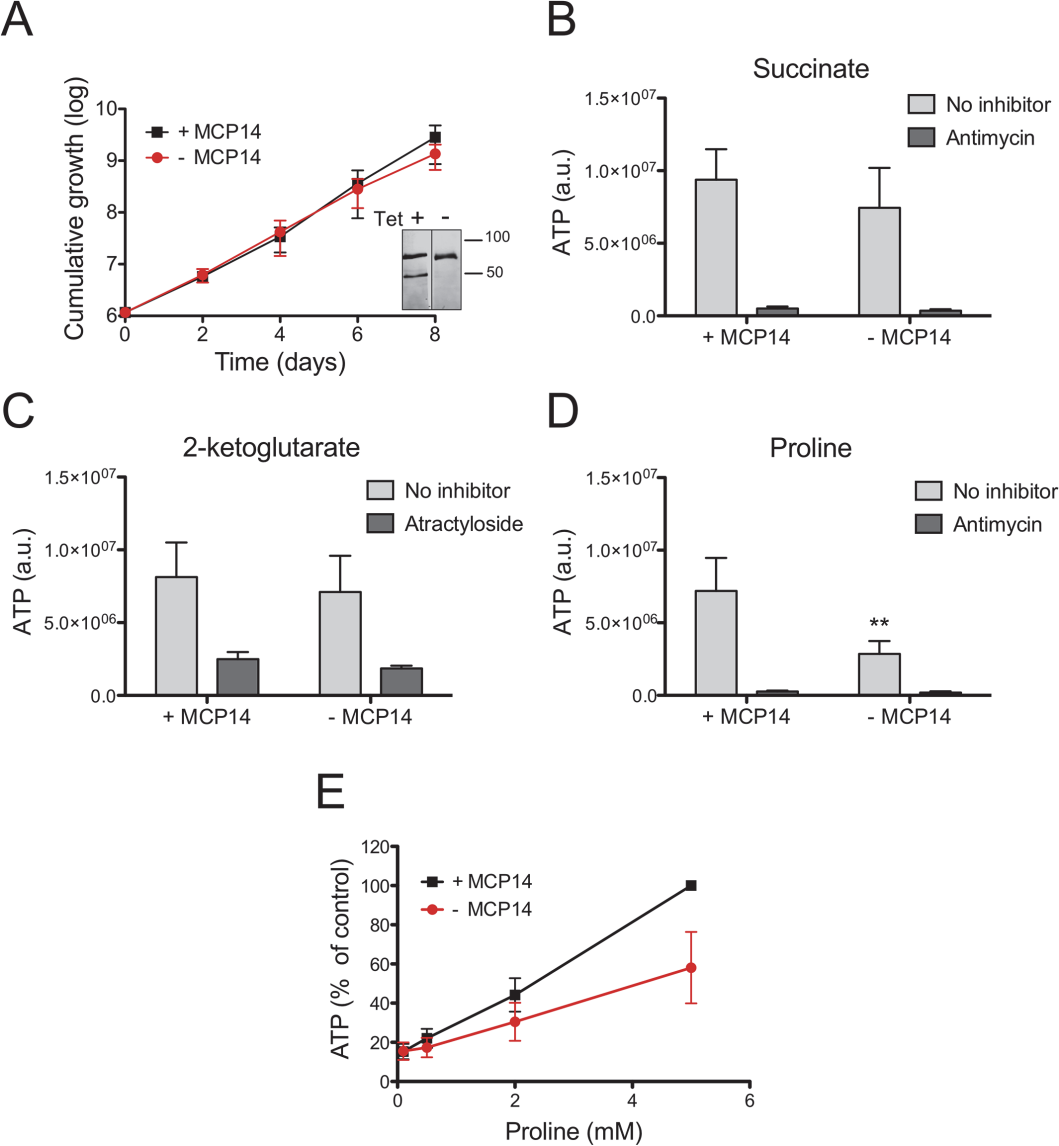
ATP production by crude mitochondria from TbMCP14 conditional knock-out procyclic forms. A) Growth curves of parasites cultured in SDM80 containing 10% normal FBS in the presence (black squares, +MCP14) or absence (circles,-MCP14) of tetracycline. Data points represent mean values ± standard deviations from three independent experiments. After 6 days of culture, parasites were permeabilized with 0.01% digitonin and crude mitochondria were isolated. The inset shows SDS-PAGE/immunoblots probed with anti-cMyc antibody to detect TbMCP14 (lower band) and anti-HSP70 antibody to demonstrate equal loading. The samples were run on the same blot and exposed for equal times (for depletion of TbMCP14 see also Fig 7D and S5 Fig). B,C,D) ATP production in isolated mitochondria was measured by addition of 67 μM ADP and 5 mM succinate (B), 2-ketoglutarate (C) or proline (D). Antimycin (2.7 μM, panels B, D) or atractyloside (43 μM, panel C) were added to inhibit complex III of the electron transport chain or ADP/ATP translocation, respectively. The bars are mean values ± SD from five independent experiments. The asterisks represent statistical difference relative to the respective controls (**p<0.01, unpaired student’s t test). E) Concentration-dependent ATP production by crude mitochondria from MCP14-expressing (black squares, +MCP14) or-depleted (red circles,-MCP14) parasites using proline as substrate. The data points have been normalized to the ATP production of control cells at the highest proline concentration (5 mM). The numbers represent mean values ± SD from four independent experiments.

It has been demonstrated that ATP production in crude mitochondria can be measured using digitonin-permeabilized trypanosome suspensions [45]. In the presence of ADP, addition of succinate to crude mitochondria results in ATP production via oxidative phosphorylation, whereas addition of 2-ketoglutarate induces ATP formation via substrate level phosphorylation [46]. We now used these conditions to measure ATP production in crude mitochondria of TbMCP14 conditional knock-out trypanosomes. Crude mitochondria were prepared from parasites cultured in SDM80 containing glucose in the presence or absence of tetracycline for 6 days to maintain or deplete, respectively, expression of TbMCP14. Under these conditions, depletion of TbMCP14 had no effect on parasite growth (Fig 10A). Crude mitochondria isolated from control and TbMCP14-depleted parasites (Fig 10A, inset) were incubated for 30 min in the absence or presence of succinate, 2-ketoglutarate or proline as substrates for ATP production. We found that depletion of TbMCP14 has no effect on ATP formation using succinate or 2-ketoglutarate as substrates (Fig 10B and 10C). In contrast, ATP production using different concentrations of proline as substrate was decreased in mitochondria from TbMCP14-depleted parasites compared to control cells (Fig 10D and 10E). The observation that proline-dependent ATP production was inhibited by antimycin (Fig 10D), an inhibitor of complex III of the electron transport chain, indicates that ATP is formed via oxidative phosphorylation. Together, these results strongly indicate that TbMCP14 is involved in metabolism of proline for energy production in mitochondria, possibly by promoting proline transport through the mitochondrial inner membrane.

### TbMCP14 has an unusual domain organization and is unique to trypanosomatids

While the N-terminal half of TbMCP14 (amino acids 1 to 161) is devoid of known motifs, the C-terminal half (amino acids 162 to 402) carries mitochondrial carrier protein (MCP) motifs of about 100 amino acids each, which are typical for transporters of the inner mitochondrial membrane (Pfam entry PF00153). Mitochondrial carriers usually possess three such motifs, whereas TbMCP14 only has two. A profile search with PF00153 against SwissProt, the manually curated section of UniProt [47], returned 1347 hits of E-value <10^–10^, of which only 24 (1.8%) possessed two MC motifs while 1302 (97%) had three. Blastp similarity searches [48] with TbMCP14 as the query returned highly significant hits (expectancy E<10^–12^) from trypanosomatids only, followed by many hits from the viridiplantae. The most similar human protein to TbMCP14 was SLC25A44, a mitochondrial carrier of unknown function [36]. A phylogenetic tree of a Muscle multiple alignment [49] of representative hits supplemented with selected human MCPs (S5 Fig) confirmed that the trypanosomatid TbMCP14 orthologues form a clearly separate clade within the mitochondrial carrier superfamily.

## Discussion

Previous studies have shown that choline analogs are potent inhibitors of choline uptake and affect PC metabolism in protozoa [25,26]. However, more recent results [33], including our own involving pulse-chase experiments using labeled choline in presence of G25 in *T*. *brucei* procyclic forms [20], indicated that inhibition of phospholipid synthesis may not be the main target of G25 in trypanosomes. Instead, these reports indicated that parasite death by choline analogs is mediated via affecting mitochondrial function. A mechanism for the uptake and site of action of the drugs was, however, not proposed. We now demonstrate that toxicity of the choline analogs G25, T4 and T3, is mediated by a member of the mitochondrial carrier family, TbMCP14. Evidence for the involvement of TbMCP14 in drug action is several fold: First, TbMCP14 was identified by an unbiased screening approach using a RNAi library in *T*. *brucei* bloodstream forms. Its down-regulation resulted in selection of parasite populations showing substantially lower susceptibilities towards the three drugs in separate screens. Second, depletion of TbMCP14 by RNAi in both bloodstream and procyclic forms increased resistance of parasites towards the drugs on average by 7-fold and 3-fold, respectively, compared to uninduced cells. Third, over-expression of a tagged form of TbMCP14 in procyclic forms resulted in hypersensitivity towards T3. Fourth, down-regulation of TbMCP14 protected bloodstream form mitochondria from drug-induced decrease in mitochondrial membrane potential.

The mitochondrial carrier family consists of a group of related proteins with conserved sequence features, i.e. three tandem repeats of about 100 amino acids, each of them with two predicted transmembrane alpha helices connected by a hydrophilic loop, and a conserved signature signal sequence motif [50,51]. Members of the mitochondrial carrier family are involved in transport of mono-, di- and tricarboxylates, co-factors like NAD^+^, FAD and coenzyme A, amino acids, and other substrates necessary for mitochondrial function [52]. In a previous report, *in silico* analysis of the *T*. *brucei* genome using conserved amino acid sequences and protein domains suggested the presence of 24 mitochondrial carrier family proteins [36]. In addition, using previously described and characterized mitochondrial carriers from yeast and humans as references, putative functions were proposed for 20 of the predicted carriers in *T*. *brucei* [36]. At present, however, biochemical data about their substrate specificities or physiological functions is only available for a few members, one of them being TbMCP5, an ATP/ADP translocator [39]. We now show that TbMCP14 belongs to a trypanosomatid-specific clade of mitochondrial carrier family proteins that shows relatively weak similarity to mitochondrial carriers of mammals, making it an interesting target for drug action and/or targeting.

Attempts to express TbMCP14 in *Xenopus laevis* oocytes or *Saccharomyces cerevisiae* to study substrate specificity were unsuccessful, possibly because the carrier mislocalized in these model expression systems. However, by generating TbMCP14 knock-out mutants in *T*. *brucei* bloodstream and procyclic forms, we were able to demonstrate that TbMCP14 is closely linked to mitochondrial energy production. Deletion of both alleles of TbMCP14 in bloodstream forms showed that the carrier is not essential for survival in this life-cycle form, but reduced parasite proliferation under standard culture conditions, i.e. in the presence of glucose. A much stronger growth defect was observed for procyclic form TbMCP14 conditional knock-out parasites. Ablation of TbMCP14 expression resulted in growth arrest. Interestingly, the time point at which parasite proliferation stopped was dependent on the availability of glucose as energy source in the culture medium. In medium containing low glucose (SDM80), procyclic form trypanosomes have been shown to switch from glucose metabolism to catabolism of amino acids, mostly proline [44,53]. Under these conditions, growth of TbMCP14-depleted parasites was clearly reduced compared to control trypanosomes cultured in the presence of standard glucose concentrations (SDM79). In line with these observations, we found that proline-dependent ATP production in crude mitochondria from TbMCP14-depleted procyclic form trypanosomes was clearly reduced compared to control mitochondria. In contrast, no changes in ATP production were observed in mitochondria after knocking out TbMCP14 when succinate or 2-ketoglutarate were used as substrates for oxidative phosphorylation or substrate level phosphorylation, respectively, demonstrating that the processes themselves were not affected by the absence of TbMCP14. Together with our observation that the levels of the proline metabolite, pyrroline-5-carboxylate, were affected in procyclic forms over-expressing TbMCP14, these results demonstrate that TbMCP14 is involved in proline-dependent energy production, possibly by acting as a mitochondrial proline carrier. Since proline-dependent ATP production in mitochondria isolated from TbMCP14-depleted cells can still be observed, TbMCP14's role in proline uptake may be indirect. Alternatively, other transporters may allow proline import into mitochondria in the absence of TbMCP14.

Our observation that depletion of TbMCP14 also affects growth of bloodstream form parasites, which don’t rely on proline as source of energy, may be explained by its ability to transport other essential metabolites. In fact, relatively broad substrate specificities have been reported for several members of the MCP family. In addition, available data indicate that mitochondrial proline dehydrogenase, and thus proline metabolism, is also important for normal growth of bloodstream forms (http://www.genedb.org/gene/Tb927.7.210). Furthermore, inhibition of proline transport by depletion of TbMCP14 may affect mitochondrial protein synthesis.

At present, it is unclear how choline analogs cross the plasma membrane of *T*. *brucei*. Previous studies in *Plasmodium* using radiolabeled members of the group of choline analogs suggested that the compounds are taken up into parasites involving an erythrocyte plasma membrane choline carrier [25,30]. Although choline is efficiently taken up by *T*. *brucei* bloodstream and procyclic forms [20], a choline carrier has not yet been identified.

## Materials and Methods

All reagents were of analytical grade and purchased from Merck (Darmstadt, Germany), Sigma-Aldrich (Buchs, Switzerland) or ICN Biomedicals (Tägerig, Switzerland). Antibiotics and fetal bovine serum (FBS) were obtained from Invitrogen (Basel, Switzerland). Dialyzed fetal calf serum (FCS) was obtained from BioConcept Amimed (Allschwil, Switzerland). DNA polymerases were purchased from Promega (Madison, USA). Primers and sequencing services were from Microsynth AG (Balgach, Switzerland). All restriction enzymes were purchased from Fermentas (Nunningen, Switzerland). [α-^32^P]-dCTP (3000 Ci/mmol) was from PerkinElmer Life Sciences (Schwerzenbach, Switzerland) and Kodak MXB and BioMax MS films were from Kodak SA (Lausanne, Switzerland).

### Trypanosomes and culture conditions

*T*. *brucei* bloodstream forms co-expressing T7 RNA polymerase and a tetracycline repressor (known as New York single-marker cells, NY-SM; [54]) were cultured at 37°C in HMI-9 containing 10% (v/v) heat-inactivated FBS. Derived clones to down-regulate or over-express TbMCP14 were cultured in the presence of 2.5 μg/ml phleomycin or 0.1 μg/ml puromycin, respectively. TbMCP14 knock-out parasites were cultured in the presence of 5 μg/ml hygromycin and 2.5 μg/ml blasticidin. *T*. *brucei* 29–13 procyclic forms [54] were cultured at 27°C in SDM79 containing 10% (v/v) heat-inactivated FBS, in the presence of 25 μg/ml hygromycin, and 15 μg/ml G418. The derived clones containing different double-stranded RNA constructs against TbMCP14 (Tb927.10.13120) were cultured in the presence of an additional 2 μg/ml puromycin. TbMCP14 conditional knock-out procyclic forms were cultivated in the presence of 5 μg/ml blasticidin, 0.2 μg/ml phleomycin and 2 μg/ml puromycin, and 1 μg/ml tetracycline to maintain expression of the ectopic copy of TbMCP14. Growth of *T*. *brucei* procyclic forms in glucose-depleted medium was studied in SDM80 [44], supplemented with 9% dialyzed (10’000 molecular weight cut-off) and heat-inactivated FCS and 1% non-dialyzed and heat-inactivated FBS.

### Drug sensitivity assays and screening of RNAi library

Susceptibility of *T*. *brucei* bloodstream forms to G25, T3 and T4 was assessed by Alamar blue assays [55]. Briefly, serial dilutions of G25, T3 or T4 starting at 10, 250 or 10 μM (from 10 mM aqueous stock solutions), respectively, were prepared in HMI-9 containing 10% (v/v) FBS in 96-well plates (100 μl final volume). Pentamidine, DB75 and diminazene aceturate were added to parasite cultures from stock solutions in DMSO (100 μM, 2.2 mM and 20 mM, respectively), isometamidium chloride was added from a 20 mM aqueous stock solution. Parasites were added to a final density of 1 × 10^4^ cells/ml. After incubation for 70 h at 37°C, 10 μl of Alamar blue solution (12.5 mg of resazurin in 100 ml PBS, composed of 137 mM NaCl, 2.7 mM KCl, 10 mM Na_2_HPO_4_, 1.76 mM KH_2_PO_4_, pH 7.2) was added to all wells and incubation was continued for another 2 h at 37°C. Fluorescence was measured using a spectromax GEMINI plate reader at 544 nm excitation, 590 nm emission and 570 nm cut-off. Drug screening using an RNAi library constructed in *T*. *brucei* bloodstream forms was performed as described before [9]. Briefly, a frozen stabilate of 3 × 10^6^ parasites was thawed in 30 ml HMI-9 supplemented with 10% FBS and daily diluted to the same density (3 × 10^6^ cells/30 ml). After three passages, RNAi was induced by addition of tetracycline to the culture. After three days, the parasite culture was split in flasks containing 10^6^ cells/10 ml medium. Drug concentrations determined to kill 98% of parasites (EC_98_) by Alamar blue assays were used for selection of resistant parasites. EC_98_ concentrations of G25, T3 or T4 were added to the culture flasks, which were incubated until resistant populations started growing. Finally, genomic DNA was extracted from resistant parasites. DNA fragments conferring resistance were amplified by PCR, cloned into PCRII-TOPO (Invitrogen) and identified by DNA sequencing.

### RNAi-mediated gene silencing, over-expression and generation of knock-out parasites

Two RNAi vectors, pALC14 and pMS14 (derivatives of pLew100 [54]), both harboring a tetracycline-inducible stem loop, were used to down-regulate TbMCP14. The pALC14 plasmid (described in [46]) has the stem loop under control of the GPEET promoter and was used to transfect procyclic forms, whereas the pMS14 plasmid [56], which is regulated by an rRNA promoter, was used to transfect bloodstream forms. TbMCP14 gene fragments were cloned into the vectors using PCR products obtained with primers 1 and 2 (S1 Table), resulting in plasmids pJPM14pc and pJPM14bs.

For over-expression in procyclic and bloodstream form trypanosomes, the TbMCP14 open reading frame (amplified using primers 3 and 4, S1 Table) was inserted into an expression vector based on pLew100 [54,57], containing a C-terminal extension encoding 3x-cMyc to allow tetracycline-inducible expression of cMyc-tagged TbMCP14, resulting in plasmid pJPM14O. The same strategy was used to induce over-expression of the truncated version of TbMCP14 except that a shorter open reading frame (amplified using primers 5 and 4, S1 Table) was inserted in the vector.

Procyclic form TbMCP14 conditional knock-out parasites were generated stepwise by i) replacing one of the endogenous alleles with a blasticidin resistance gene by homologous recombination, ii) inserting an ectopic copy of TbMCP14, C-terminally tagged with cMyc, and iii) replacing the remaining endogenous allele of TbMCP14 by a phleomycin resistance gene. The corresponding vectors were generated as follows; first, a fragment of 400 nt from the 5’-flanking region of TbMCP14 was amplified using primer 6, having an XhoI restriction site, and primer 7, having a HindIII restriction site (Suppl. S1 Table). Second, a fragment of 458 nt from the 3’-flanking region of TbMCP14 was amplified using primers 8 and 9 (Suppl. S1 Table). Subsequently, the two fragments were inserted into vector pKO^blast^ [58], which contains the procyclin EP1-EP2 intergenic region, a blasticidin resistance gene, and the tubulin intergenic region, resulting in vector pJPM14KO^blast^. The blasticidin resistance gene was then replaced by a phleomycin resistance gene using the flanking restriction sites AscI and PacI, resulting in vector pJPM14KO^phleo^.

Plasmid extraction was performed using Qiagen Plasmid Midi Kit (Qiagen, Hilden, Germany) according to the manufacturer’s instructions. Before transfection of *T*. *brucei* parasites, pLew-based plasmids were linearized with *Not*I while pKO plasmids were digested with XhoI/NotI to release linear fragments containing the respective resistance genes flanked by sequences for homologous recombination with TbMCP14.

### Stable transfections of trypanosomes

*T*. *brucei* procyclic and bloodstream forms were harvested at mid-log phase, washed once in buffer (132 mM NaCl, 8 mM KCl, 8 mM Na_2_HPO_4_, 1.5 mM KH_2_PO_4_, 0.5 mM magnesium acetate, 0.09 mM calcium acetate, pH 7.0) and resuspended in fresh buffer at a density of 8 × 10^7^ cells/ml. Subsequently, 440 μl of parasite suspension were mixed with 10–15 μg of digested plasmids and transferred to a 0.2-cm pulse cuvette (Bio-Rad). Electroporation was conducted with a BTX Electroporation 600 system (Axon Lab, Baden, Switzerland) with one pulse (1.5 kV charging voltage, 2.5 kV resistance, 25 microfarads capacitance timing, and 186 resistance timing). Cells were immediately inoculated in 10 ml of procyclic or bloodstream form medium. Dilutions were plated into 24-well plates and after 24 h selected for antibiotic resistance. Clones were obtained by limiting dilution.

### mRNA analyses

Total RNA was isolated using the SV Total RNA Isolation System (Promega), following the manufacturer’s instructions. cDNA was synthesized from total RNA (0.1–0.5 μg) using SuperScript II reverse transcriptase (Invitrogen).

For Northern blotting, total RNA (10–15 μg) was separated on formaldehyde-agarose gels (1% agarose, 2% formaldehyde in 20 mM Mops, pH 7.0, containing 8 mM sodium acetate and 1 mM EDTA) and transferred to Amersham Hybond-N^+^ nylon membranes (GE Healthcare, Buckinghamshire, UK). The PCR products used to construct the RNAi stem loop vectors served as templates to make the [^32^P]-labeled probes by random priming, using Prime-a-Gene Labeling System (Promega). Hybridization was performed overnight at 60°C in hybridization buffer containing 7% (w/v) SDS, 1 mM EDTA, 0.5 M Na_2_HPO_4_, 24 mM H_3_PO_4_, pH 7.2, and the membrane was analyzed by autoradiography using BioMax MS film and a TransScreen-HE intensifying screen. Ribosomal RNA was visualized on the same formaldehyde-agarose gel by ethidium bromide staining to control for equal loading.

### Fluorescence microscopy

Parasites (10^6^ in 100 μl) were allowed to adhere to a microscope slide for 10 min, fixed with 4% paraformaldehyde in PBS, washed with PBS, and permeabilized with 0.2% (w/v) Triton X-100 in PBS. After incubation in PBS containing 2% bovine serum albumin (blocking buffer) for 30 min, primary antibody in blocking solution was added for 45 min. Antibodies used were mouse monoclonal anti-cMyc 9E10 (Santa Cruz Biotechnology, Heidelberg, Germany) and rabbit anti-VDAC antiserum at dilutions of 1:250 and 1:1000, respectively. After washing with PBS, the corresponding secondary fluorophore-conjugated antibodies, goat anti-mouse Alexa Fluor 594 and goat anti-rabbit Alexa Fluor 488 (Invitrogen), respectively, at dilutions of 1:100 in blocking solution, were added for 45 min. Free antibody was removed by washing with PBS and cells were mounted with Vectashield containing 4,6-diamidino-2-phenylindole (DAPI; Vector Laboratories). The slides were analyzed using a Leica SP2 microscope equipped with a 100 × oil objective. Photographs were acquired with Leica LAS AF Version 2.1.0 software (Leica Microsystems).

### Flow cytometry

*T*. *brucei* bloodstream forms were incubated in the presence of 80–350 nM G25 for 24 h. Aliquots of 0.5 ml were taken to measure mitochondrial membrane potential (ΔΨ_m_) and cell permeability by propidium iodide (PI) staining. The ΔΨ_m_ was measured by adding 25 nM tetramethylrhodamine ethyl ester (TMRE) to bloodstream form cultures. After 30 min of incubation at 37°C, parasites were washed with and resuspended in PBS, and immediately analyzed by flow cytometry (FACScan BD, equipped with Cytek solid state laser) using the FL2-channel detector. The geometrical mean values of 10’000 gated events were normalized to control samples. In control cultures, 50 μM carbonyl cyanide *m*-chlorophenyl hydrazone was added to disrupt ΔΨ_m_. For evaluation of cell permeability, 10 μg/ml of PI was added to parasite cultures and incubated for 10 min at 37°C, protected from light. Subsequently, 0.5 ml of culture was transferred to FACS tubes and fluorescence was measured using FL3-channel detector. Ten thousand gated events were separated into two areas—according to fluorescence intensity—as follows: the fluorescence intensity of a sample containing digitonin-permeabilized parasites was measured and referred to as PI-positive (PI+). Fluorescence intensities lower than this value were considered PI-negative (PI-).

### Preparation of crude mitochondria and ATP production assays

A crude mitochondrial fraction from TbMCP14 conditional knock-out parasites cultured in glucose-depleted medium (SDM80) supplemented with 10% heat-inactivated FBS were prepared as described before [45]. Briefly, 10^8^ parasites were collected by centrifugation and washed once in cold sodium phosphate buffer (150 mM Tris-HCl pH 7.9, 20 mM NaH_2_PO_4_ and 20 mM glucose). The cell pellet was resuspended in 0.5 ml SoTE (0.6 M sorbitol, 20 mM Tris-HCl, pH 7.5, and 2 mM EDTA) and combined with 0.5 ml of 0.02% (w/v) digitonin in SoTE. After 5 min of incubation on ice, the suspension was centrifuged at 5’500 × *g* and the remaining pellet (mitochondrial suspension) was resuspended in 750 μl of assay buffer (20 mM Tris-HCl, pH 7.4, 15 mM KH_2_PO_4_, 0.6 M sorbitol, 10 mM MgSO_4_, 10 mg/ml fatty-acid-free bovine serum albumin).

ATP production assays were done as described [59]. Briefly, 5 mM succinate, 5 mM 2-ketoglutarate or different concentrations of proline, together with 67 μM ADP, were added to 71.5 μl of mitochondrial suspension. After incubation at room temperature for 30 min, the reaction was stopped and the ATP concentration was determined using ATP Bioluminescence Assay Kit CLS II (Roche, Basel, Switzerland). Inhibitors were pre-incubated with mitochondrial suspension for 10 min on ice and used at the following final concentrations: atractyloside (43 μM) and antimycin (2.7 μM).

### Metabolomic analysis

Parasites (5 × 10^7^ cells) collected from cultures grown to mid log phase were harvested and quenched and metabolites extracted in 100 μl of chloroform/methanol/water (1:3:1, by vol) as previously described in [60]. HPLC using a ZIC-pHILIC column (150 mm × 4.6 mm, 5 μm column, Merck Sequant and a Dionex UltiMate 3000 RSLC system (Thermo, Hemel Hempstead, UK) with metabolite masses identified using a Thermo Orbitrap Exactive (Thermo Fisher Scientific, Hemel Hempstead, UK) operated in polarity switching mode with lock-mass correction applied to enhance calibration stability.

XCMS software [61] was used for untargeted peak detection and mzMatch.R [62] for peak matching and annotation of related peaks. The IDEOM software package [63] was used to identify metabolites either through matching accurate masses and retention times of authentic standards (Metabolomics Standards Initiative confidence level 1) or using predicted retention times using a previously validated model [64] (Metabolomics Standards Initiative confidence level 2) if authentic standards were not available.

### Bioinformatic analyses

Profile searches were performed with the command line version of HMMer 3.01 [65] and the results were parsed with ad hoc Perl scripts. SwissProt release 2014_05 was downloaded from ftp.uniprot.org. Blast searches were carried out on blast.ncbi.nlm.nih.gov. Muscle multiple alignments and Neighbor-Joining trees were done with amino acid sequences on Mega5 [66], using default parameters and the JTT substitution model. GenBank accession numbers of the (full-length) sequences of S5 Fig are the following: *T*. *vivax*, 340057877; *T*. *grayi*, 686632047; *T*. *cruzi*, 407846745; *T*. *rangeli*, 554941519; *L*. *donovani*, 398013843; *L*. *major*, 157867905; *L*. *braziliensis*, 154335581; *Phytomonas* sp., 588319594; *Strigomonas culicis*, 528241051; *Angomonas deanei*, 528250442; *Ricinus communis*, 255580342; *Glycine max*, 356568805; *Oryza sativa*, 115455415; *Medicago truncatula*, 657381127; *Vitis vinifera*, 359488385; SLC25AA1, 13436407; SLC25AA4, 178659; SLC25AA29, 119602101; SLC25AA32, 18256909; SLC25AA36, 119599418; SLC25AA44, 14250748.

## Supporting Information

S1 Table Oligonucleotides used in the study. (PDF)

S1 Fig Viability of *T*. *brucei* bloodstream forms towards G25 after modulation of TbMCP14 expression. (PDF)

S2 Fig Sensitivity of *T*. *brucei* procyclic forms over-expressing TbMCP5 towards T3 and pentamidine. (PDF)

S3 Fig TbMCP14-dependent sensitivity of *T*. *brucei* bloodstream forms towards G25. (PDF)

S4 Fig SDS-PAGE/immunoblot analysis of lysates of *T*. *brucei* procyclic form TbMCP14 conditional null mutants. (PDF)

S5 Fig Neighbour joining tree of mitochondrial carrier proteins (MCPs). (PDF)
