# Supplementary material for: An Atypical Mitochondrial Carrier That Mediates Drug Action in Trypanosoma brucei
Source: PLoS Pathog. 2015 May 6;11(5):e1004875. doi: 10.1371/journal.ppat.1004875 (PMC4422618; doi:10.1371/journal.ppat.1004875)
Supplement: S1 Table — (PDF) [file ppat.1004875.s001.pdf]

S1 Table. Oligonucleotides used in the study.

Primer number (name)	*Sequence*
Primer 1 (TbMCP14-F)	5'- GCCCAAGCTTGGATCCGAGGGGTCTTCGGGCTATTTA -3'
Primer 2 (TbMCP14-R)	5'- TGGCTCTAGACTCGAGCAGAAGCCCAGCGTAGAGAC -3'
Primer 3 (TbJPM14O-F full-length)	5'- ACGCAAGCTTATGAGTATCACCAACATCGCTG -3'
Primer 4 (TbJPM14O-R)	5'- CGCGGATCCTATTTGTTTCCAGAGTTCTCGGTC -3'
Primer 5 (TbJPM14O-F truncated)	5'- ACGCAAGCTTATGCTACGAACGTTGCTGCTG -3'
Primer 6 (MCP14KO-5'_fw)	5'- CCCCTCGAGAACGGATTTATGGAGTTTATTTTTC -3'
Primer 7 (MCP14KO-5'_rv)	5'- GCCCAAGCTTCTAACTTAATGTGAGGACGTC -3'
Primer 8 (MCP14KO-3'_fw)	5'- TGGCTCTAGAAGGAGAAATGGTGTGGTACAC -3'
Primer 9 (MCP14KO-3'_rv)	5'- CCCGCGGCCGCTGGTGATTAAACCTCACTTTATC -3'
Primer 10 (MCP14qPCR_fw)	5'- CGAAACCTCCATCCGTATTC -3'
Primer 11 (MCP14qPCR_rv)	5'- CCACGATAAATAGCCCGAAG -3'
Primer 12 (Splice leader)	5'- CGCTATTATTAGAACAGTTTCTGTAC -3'
