# Supplementary material for: An Atypical Mitochondrial Carrier That Mediates Drug Action in Trypanosoma brucei
Source: PLoS Pathog. 2015 May 6;11(5):e1004875. doi: 10.1371/journal.ppat.1004875 (PMC4422618; doi:10.1371/journal.ppat.1004875)

S1 Figure. Sensitivity of *T. brucei* procyclic forms over-expressing TbMCP5 towards T3 and pentamidine. *T. brucei* 427 procyclic forms were transfected with a plasmid encoding N-terminally cMyc-tagged TbMCP5 (generated from a TbMCP5 plasmid obtained from F. Voncken, University of Hull, U.K.) and clones were obtained by limited dilution in presence of 25 µg/ml hygromycin. Panel A: Expression of TbMCP5 in clones 1 and 2 was analyzed by SDS-PAGE/immunoblotting using anti-cMyc antibody. The parental cell line (427) is shown as negative control. Equal protein loading was verified with anti-EF1a antibody. Molecular mass markers are indicated in the left margin. Panels B,C) Alamar blue assays to determine the sensitivity of TbMCP5-expressing parasite clones 1 and 2 (indicated as red circles and blue squares, respectively) towards T3 (panel B) and pentamidine (panel C). Black squares represent parental (427) parasites. The data points represent mean values ± SEM of triplicate determinations from single experiments. The insets on the right show the EC_{50} values of the respective curves.

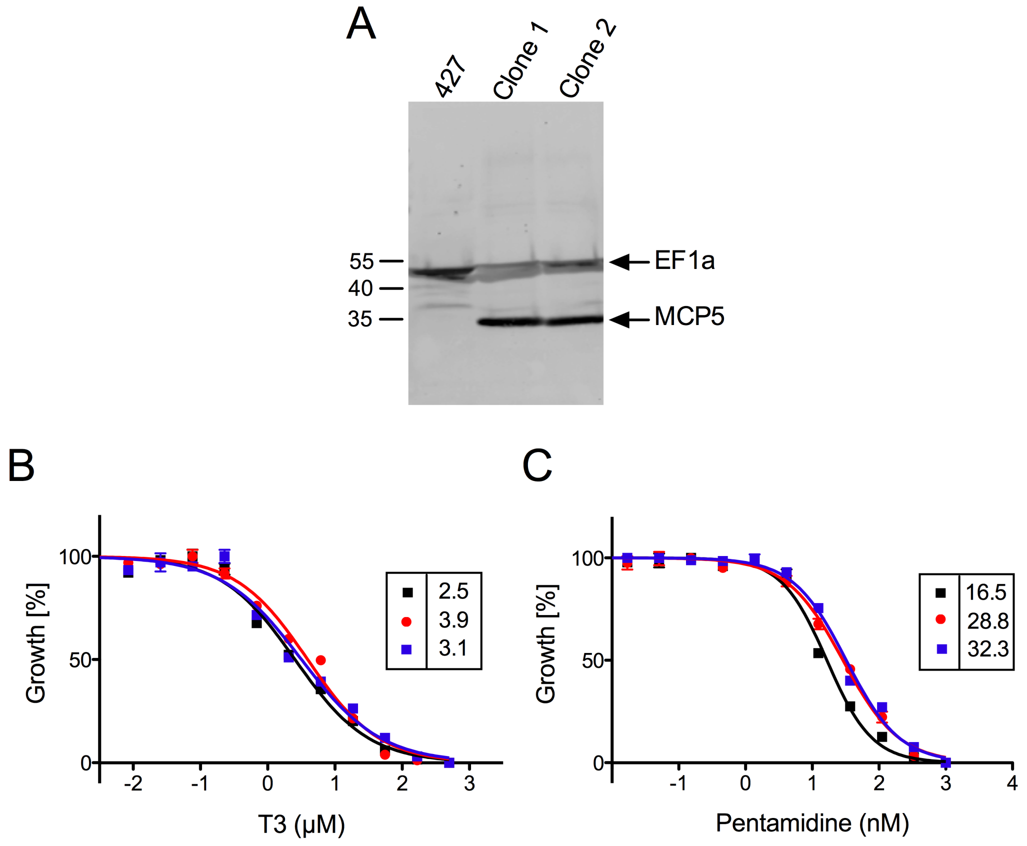

Supplement: S1 Fig — (PDF) [file ppat.1004875.s002.pdf]
