# Supplementary material for: An Atypical Mitochondrial Carrier That Mediates Drug Action in Trypanosoma brucei
Source: PLoS Pathog. 2015 May 6;11(5):e1004875. doi: 10.1371/journal.ppat.1004875 (PMC4422618; doi:10.1371/journal.ppat.1004875)

S2 Figure. Viability of *T. brucei* bloodstream forms towards G25 after modulation of TbMCP14 expression. Parasites cultured for three days in the absence (left panels) or presence (right panels) of tetracycline to induce over-expression (upper panels) or down-regulation (lower panels) of TbMCP14 were treated with different concentrations of G25 for 24 (black squares), 48 (red circles) or 72 h (green triangles). Digitonin-permeabilized cells were used as a positive control of propidium iodide (PI) staining (PI positive area of histogram, see Materials and Methods section). The results from one of two independent experiments are shown.

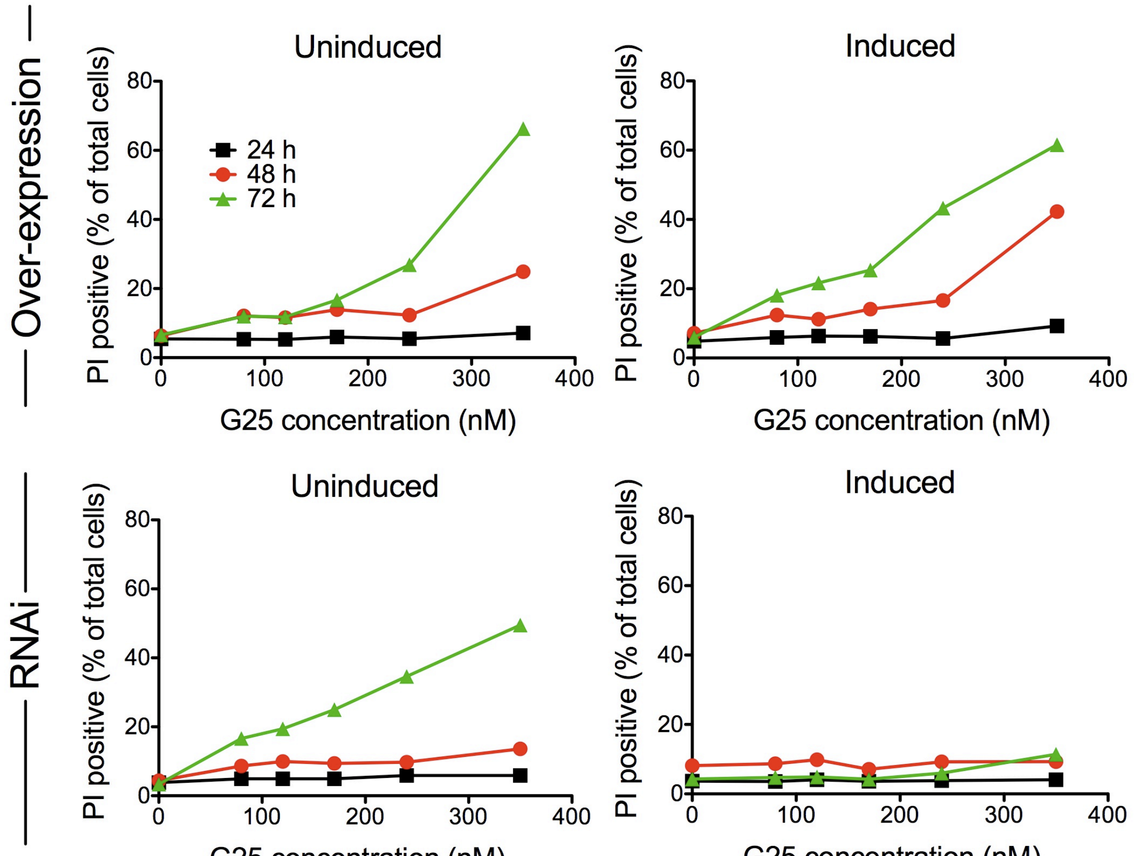

Supplement: S2 Fig — (PDF) [file ppat.1004875.s003.pdf]
