# Supplementary material for: An Atypical Mitochondrial Carrier That Mediates Drug Action in Trypanosoma brucei
Source: PLoS Pathog. 2015 May 6;11(5):e1004875. doi: 10.1371/journal.ppat.1004875 (PMC4422618; doi:10.1371/journal.ppat.1004875)

S3 Figure. TbMCP14-dependent sensitivity of *T. brucei* bloodstream forms towards G25. Parasites cultured for three days in the absence (control) or presence (tet) of tetracycline to induce down-regulation (left panels) or over-expression (right panels) of TbMCP14 were treated with different concentrations of G25 for 24 h. Percentages of propidium iodide impermeable (PI-) or permeable (PI+) parasites are shown. Digitonin-permeabilized cells were used as a positive control of PI staining (PI+ area of histogram, see Materials and Methods). The results from one of two independent experiments are shown.

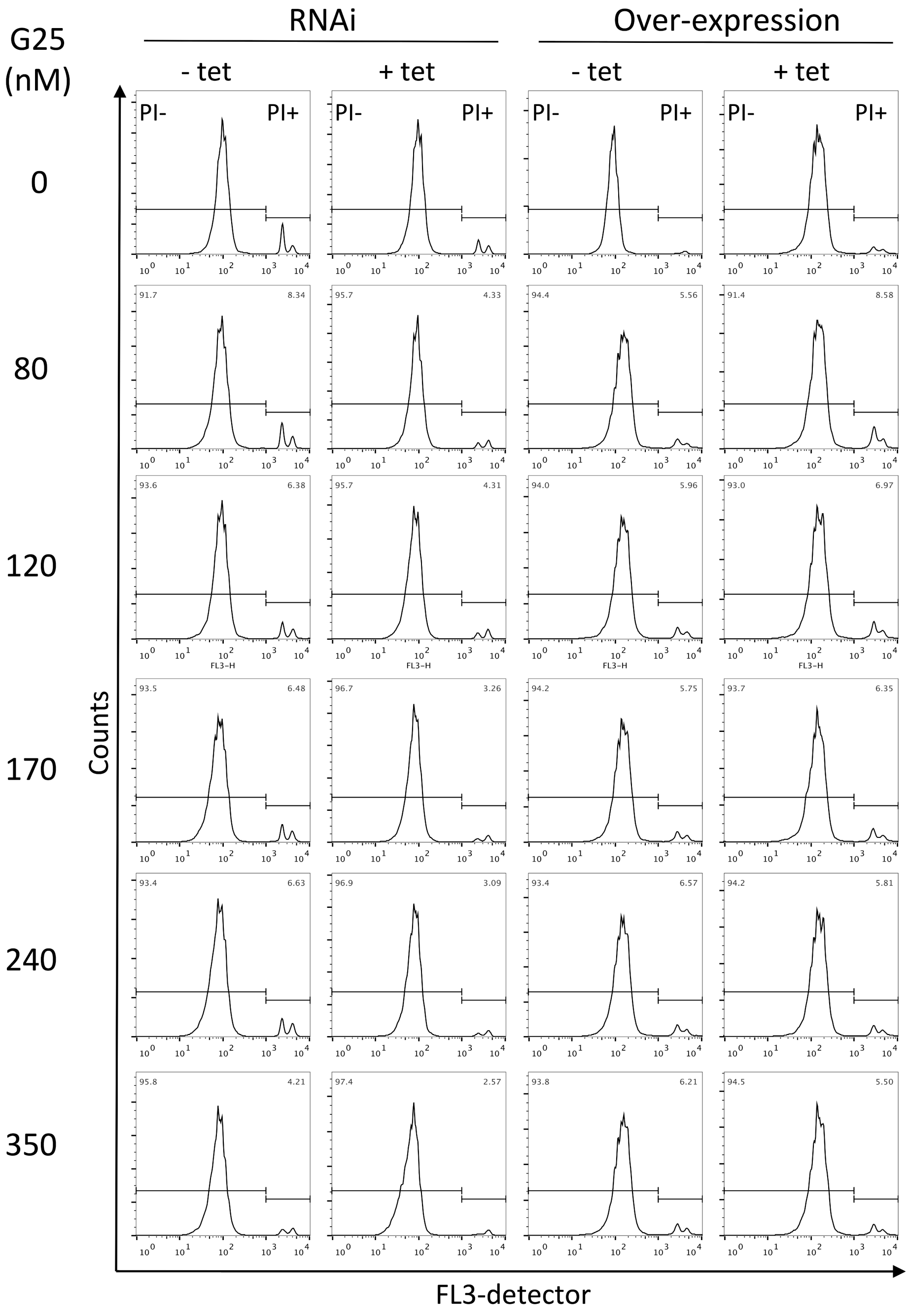

Supplement: S3 Fig — (PDF) [file ppat.1004875.s004.pdf]
