# Supplementary material for: An Atypical Mitochondrial Carrier That Mediates Drug Action in Trypanosoma brucei
Source: PLoS Pathog. 2015 May 6;11(5):e1004875. doi: 10.1371/journal.ppat.1004875 (PMC4422618; doi:10.1371/journal.ppat.1004875)

S4 Figure. SDS-PAGE/immunoblot analysis of lysates of *T. brucei* procyclic form TbMCP14 conditional null mutants grown in 0.15 mM (- glucose) or 5.5 mM (+ glucose) glucose. Parasites were cultured in the presence (+) or absence (-) of tetracycline (Tet) for indicated times. cMyc-tagged TbMCP14 (migrating at approximately 48 kDa) was visualized with anti-cMyc antibody. HSP70 (migrating at approximately 60 kDa) visualized with anti-HSP70 antibody was used as loading control. Molecular mass markers are indicated in the left margin.

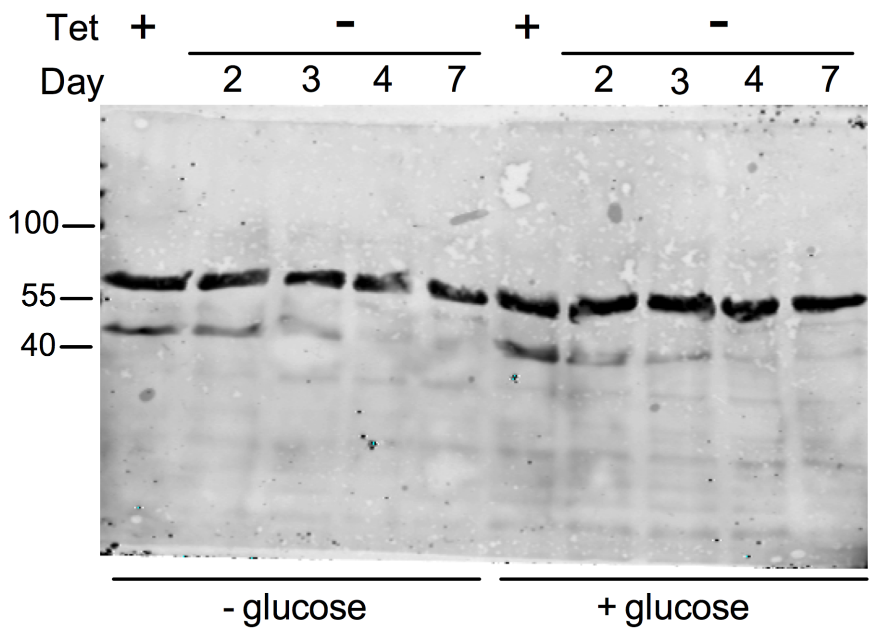

Supplement: S4 Fig — (PDF) [file ppat.1004875.s005.pdf]
