# Supplementary material for: An Atypical Mitochondrial Carrier That Mediates Drug Action in Trypanosoma brucei
Source: PLoS Pathog. 2015 May 6;11(5):e1004875. doi: 10.1371/journal.ppat.1004875 (PMC4422618; doi:10.1371/journal.ppat.1004875)

S5 Figure. Neighbour joining tree of mitochondrial carrier proteins (MCPs) from trypanosomatids (black) and plants (green) that are most similar to TbMCP14 as determined by Blastp, supplemented with selected human MCPs (purple). SLC25A44 has been identified as the closest human relative to TbMCP14 [36]. Bootstrap values (grey) are percent positives of 1'000 rounds. The scale bar indicates mutations per site.

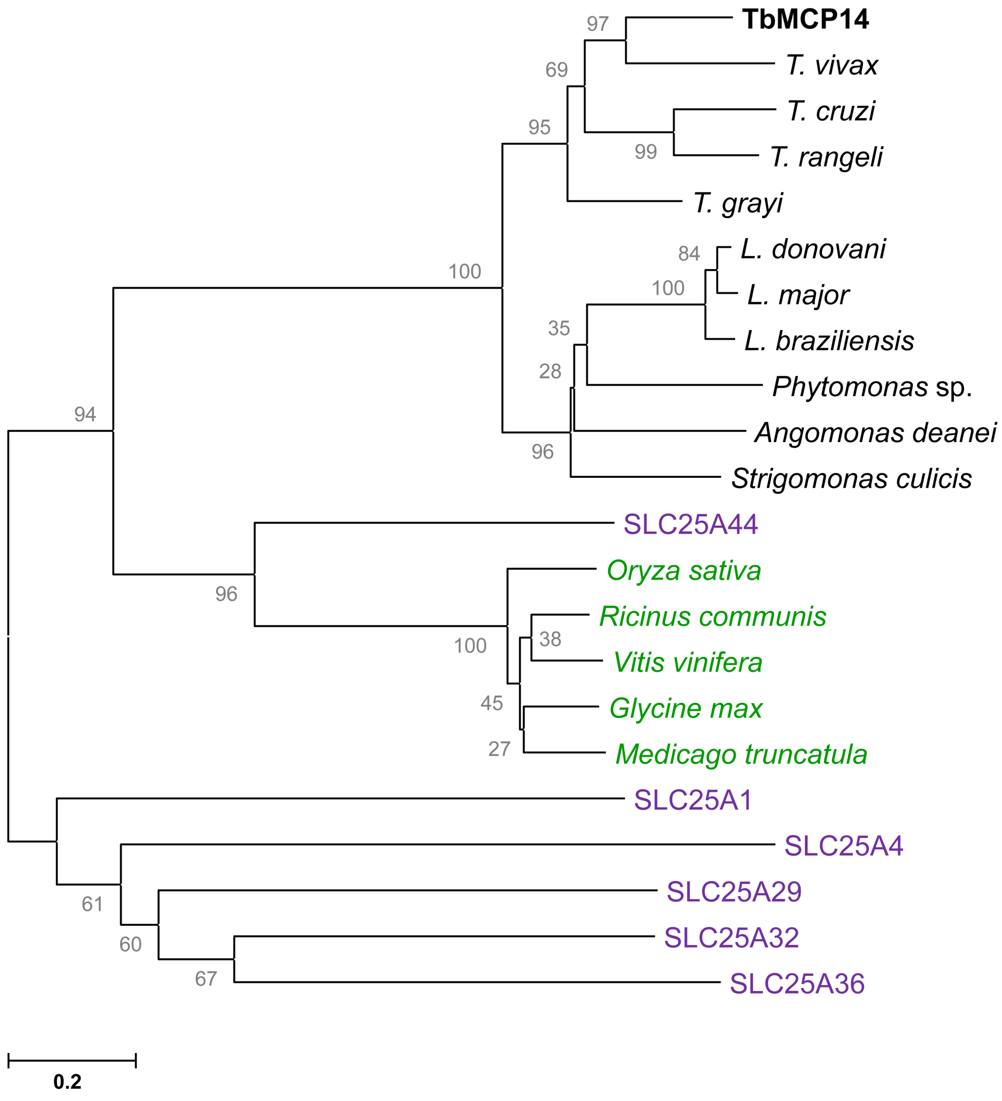

Supplement: S5 Fig — (PDF) [file ppat.1004875.s006.pdf]
